# Dichloroacetate nanoparticles and doxorubicin combinatorial treatment augment the hepato-renal function in Ehrlich ascites carcinoma cells

**DOI:** 10.1186/s13104-025-07534-3

**Published:** 2025-11-13

**Authors:** Amira T. Khattab, Mai M. El-Keiy, Doha M. Beltagy, Maha M. Salem

**Affiliations:** 1 Biochemistry Department, Faculty of Science, Damanhour University, Damanhour, 22514 Egypt; 2 Biochemistry Division, Chemistry Department, Faculty of Science, Tanta University, Tanta, 31257 Egypt

**Keywords:** Dichloroacetate nanoparticles, Doxorubicin, Combination therapy, Hepato-renal, EAC

## Abstract

**Objectives:**

Cancer cells are addressed through conventional chemotherapy, resulting in tumour resistance and systemic toxicities affecting organ functions. Nanoparticle (NPs) represent a promising approach to improve chemotherapeutic efficacy and reduce adverse effects. This study aims to improve hepato-renal function by dichloroacetate nanoparticles (DCA-PNPs) and doxorubicin (Dox) combinatorial treatment in Ehrlich ascites carcinoma (EAC) model.

**Results:**

Dichloroacetate nanoparticles characterizations showed effective drug encapsulation, optimal particles size, morphology, and distribution. Biochemical analysis showed normalized protein content, improved lipid profile, enhanced liver, kidney functions, antioxidant activity, and decreased oxidative-stress with Dox/DCA-PNPs combination treatment, indicating that NPs-based therapy enhanced therapeutic outcomes and minimized systemic toxicity via mitigated Dox side effects and maintained organ's function. This study elucidates that Dox/DCA-PNPs combination therapy provides a more effective strategy for EAC hepatorenal function improvements.

**Graphical Abstract:**

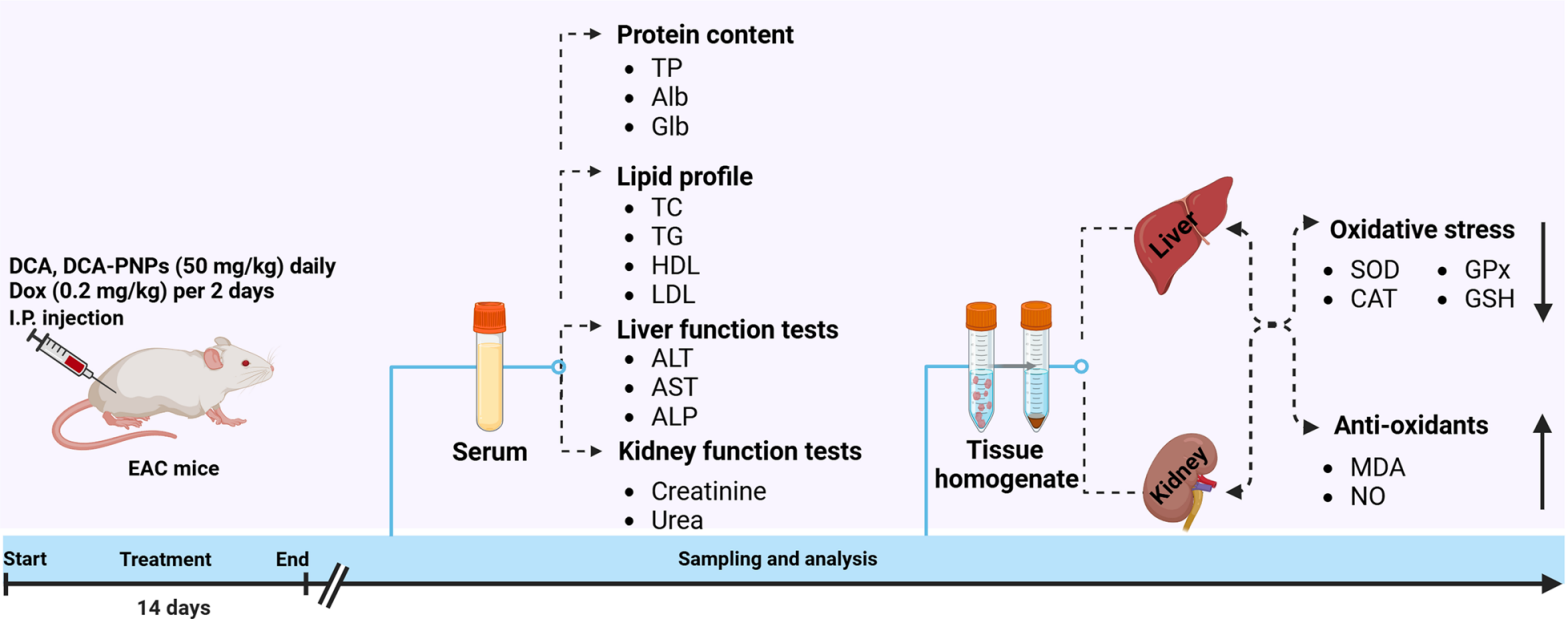

**Supplementary Information:**

The online version contains supplementary material available at 10.1186/s13104-025-07534-3.

## Introduction

Cancer is a leading cause of mortality worldwide, with breast cancer prevalent among women [1]. Traditional cancer treatments, like chemotherapy, often cause extensive organ effects and significant toxicity [2]. The Ehrlich ascites carcinoma (EAC) model is used for breast cancer research [3, 4]. Doxorubicin (Dox), a breast cancer treatment agent, which is limited by dose-dependent toxicity, including bone marrow suppression and cardiac injury [5]. Nanoparticle (NPs)-based therapy shows reduced systemic adverse effects and improved efficacy [6]. Polymeric NPs enhance efficacy and reduce toxicity in breast cancer, concentrating drugs in tumour tissues while protecting healthy organs [7, 8]. A promising strategy combines chemotherapeutic agents with metabolic modulators or antioxidants [9]. Dichloroacetate (DCA) acts as a metabolic regulator, shifting from glycolysis to oxidative phosphorylation in cancer cells to enhance treatment efficacy [10, 11]. The rationale for Dox/DCA-PNPs therapy underpins and aims to enhance anticancer effects while reducing toxicity [12, 13]. NPs maximize drug loading capacity, lowered toxic effect, improved efficiency and targeting of the tumor by showing reduced hepatotoxicity, renal toxicity, and cardiotoxicity against free Dox, in different models [14, 15]. This study examines the effect of Dox/DCA-PNPs combinatorial treatment in EAC cells, targeting cancer cells while reducing systemic toxicity.

## Materials and methods

### Chemicals and drugs

Doxorubicin hydrochloride (Dox) (98.0–102.0% HPLC, Mw 579.98, cat. no. D1515), sodium dichloroacetate (DCA) (≥ 98%; cat. no. 347795), poly D, L-lactic-co-glycolide (PLGA) (L: D 50:50, Mw 45,000; Cat. no. 805726), and polyvinyl alcohol (PVA) (80–90% hydrolyzed, Mw 30,000–70,000, Cat. no. P8136) were from Sigma-Aldrich (Germany). Bio Diagnostic company (Egypt) supplied L-malondialdehyde (MDA) (cat. no. MD 25 29), nitric oxide (NO) (cat. no. 25 33), superoxide dismutase (SOD) (cat. no. SD 25 21), catalase (CAT) (cat. no. CA 25 17), glutathione (GSH) (cat. no. R 2511), and glutathione peroxidase (GPx) (cat. no. GP 2524) kits. Vitro scient-Diagnostics (Egypt) provided total protein (TP) (cat. no. TP 20 20), albumin (Alb) (cat. no. AB 10 10), creatinine (Create) (cat. no. CR 12 50), urea (cat. no. UR 21 10), alkaline phosphatase (ALP) (cat. no. AP 10 20), alanine transaminase (ALT) (cat. no. AL 10 31), aspartate transaminase (AST) (cat. no. AS 10 61), triglycerides (TG) (cat. no. TR 20 30), total cholesterol (TG) (cat. no. CH 12 20), HDL-C (cat. no. CH 12 30), and lactate dehydrogenase (LDH) (cat. no. EC 1.1.1.27). All chemicals used were high grades.

### Preparation of DCA-PNPs

Single emulsion-evaporation technique used to synthesize DCA-PNPs using acetone (PLGA solvent) and PVA (stabilizer). PVA/H_2_O solution (2.5%) prepared by magnetic stirring (2000 rpm/1 h./45 °C). PLGA/DCA/acetone solution (1%) centrifuged (1000 rpm/5 min./room temperature), Then stirred (2000 rpm/6 h./room temperature) for evaporation, centrifuged (15,000 rpm/30 min./4 °C), dissolved in 5 mL H_2_O and lyophilized (24 h) for powder formation. Process adjusted per concentration, temperature and volatizing time according to [16].

### Characteristic features of DCA-PNPs

#### Efficiency of encapsulation and capacity for drug loading

DCA amount quantified first. Encapsulation efficiency (%EE) and loading capacity (%DL) determined by centrifugation (15,000 rpm/ 15 min./4 °C). The supernatant's concentrations measured against HPLC–UV analysis (λmax = 265 nm), %EE and %DL calculated according to [16].

#### Scanning electron microscope (SEM)

DCA-PNPs size and morphological features were examined by SEM through ion sputtering and random scanning via coating DCA-PNPs with a linked metallic stub to SEM [17].

#### Transmission electron microscope (TEM)

DCA-PNPs morphology was examined using JEOL JEM-2100 TEM and Gatan software. Diluted samples on copper grids were deposited in ethanol for examination [18].

### Experimental animals and design

Female CD1 mice from Alexandria University were acclimatised for one week at 23–25 ºC, moisture (53 ± 4%), and 12-h light–dark cycles with water and feed ad libitum. Research followed protocol (IACUC-SCI-TU-0210). Ehrlich ascites carcinoma cells were purchased from the National Cancer Institute, Cairo University and submerged in phosphate buffer saline. Cell viability was assessed via trypan blue [19, 20], with 0.5 × 10^6^ cells/mouse for i.p. inoculating [21]. Mice were divided into 10 groups (n = 7). Gp 1 served as control. Groups 2–4 received DCA (50 mg/kg/day), DCA-PNPs (50 mg/kg/day) [22], and Doxorubicin (20 mg/kg thrice weekly), [23]. Groups 5–10 were inoculated with EAC cells and received Dox, DCA, DCA-PNPs, Dox/DCA, and Dox/DCA-PNPs for 14 days. Mice were anaesthetised with sodium barbiturate (300 mg/kg) i.p. [24], that ensures rapid and deep anesthesia, minimizing any potential distress [25]. Once the animals were fully unconscious, as confirmed by the absence of pedal reflex, euthanasia was performed via cervical dislocation [26]. This two-step process was chosen to ensure a humane and efficient method of euthanasia in accordance with ethical guidelines for animal research no. (IACUC-SCI-TU-0210). Serum collected, and tissues preserved at − 20 °C for analysis.

### Biochemical analysis

Serum ALT and AST activities were determined colorimetrically according to Henley and Pollard [27, 28]. ALP activity was measured using the phenyl phosphate method [29], and LDH activity was assayed following the IFCC l-lactate: NAD⁺ oxidoreductase method [30]. Urea and creatinine concentrations were determined by the diacetyl monoxime method [31]**,** and the Jaffé reaction [32], respectively. TP was quantified by the pyrogallol red–molybdenum method [33]**,** and Alb was determined by the BCG dye-binding method [34]. Glb was calculated as the difference between TP and Alb [35]. Serum TC and TG were determined enzymatically following Allain et al. [36] and Fossati and Prencipe [37], while HDL-C was measured by the polyanion precipitation method [38]; LDL-C was calculated using the Falholt equation [39]. Hepatic and renal MDA, NO, and GSH levels were determined by the TBARS method [40], Griess reaction [41], and the method of Paglia and Valentine [42], respectively. SOD activity was measured by the NAD(P)H oxidation method [43], CAT activity by the Aebi method [44], and GPx activity following Paglia and Valentine [42].

### Statistical analysis

Results are expressed as the mean with standard error (SE). Statistics were conducted using GraphPad Prism 6. Two-way analyses of variance (ANOVA) were utilised to evaluate statistical significance. Comparative analyses were performed among groups to illustrate significant effects of treatment conditions. A (p ≤ 0.05) was statistically significant.

## Results

### Characterizations of NPs

#### Efficiency of encapsulation and capacity for drug loading of DCA-PNPs

DCA-PNPs calibration curve with 4 samples (S1 to S4) at 0.57, 0.86, 1.22, 1.67 gm/mL concentrations, respectively (Supplementary Fig. 1A) showed %EE of 97.29 ± 1.11% and %DL of 99.74 ± 0.18% (Supplementary Fig. 1B).

#### Scanning electron microscope of DCA-PNPs

SEM detected DCA-PNPs, arranged in a singular layer and exhibiting a circular morphology (Supplementary Fig. 2).

#### Transmission electron microscope of DCA-PNPs

TEM confirmed that DCA-PNPs displayed a more distinct structural configuration, resembling smooth spheres surfaces and a mean diameter of 38.58 ± 3.87 nm (Supplementary Fig. 3).

### DCA-PNPs improve liver and kidney functions levels

**Fig. 1:**
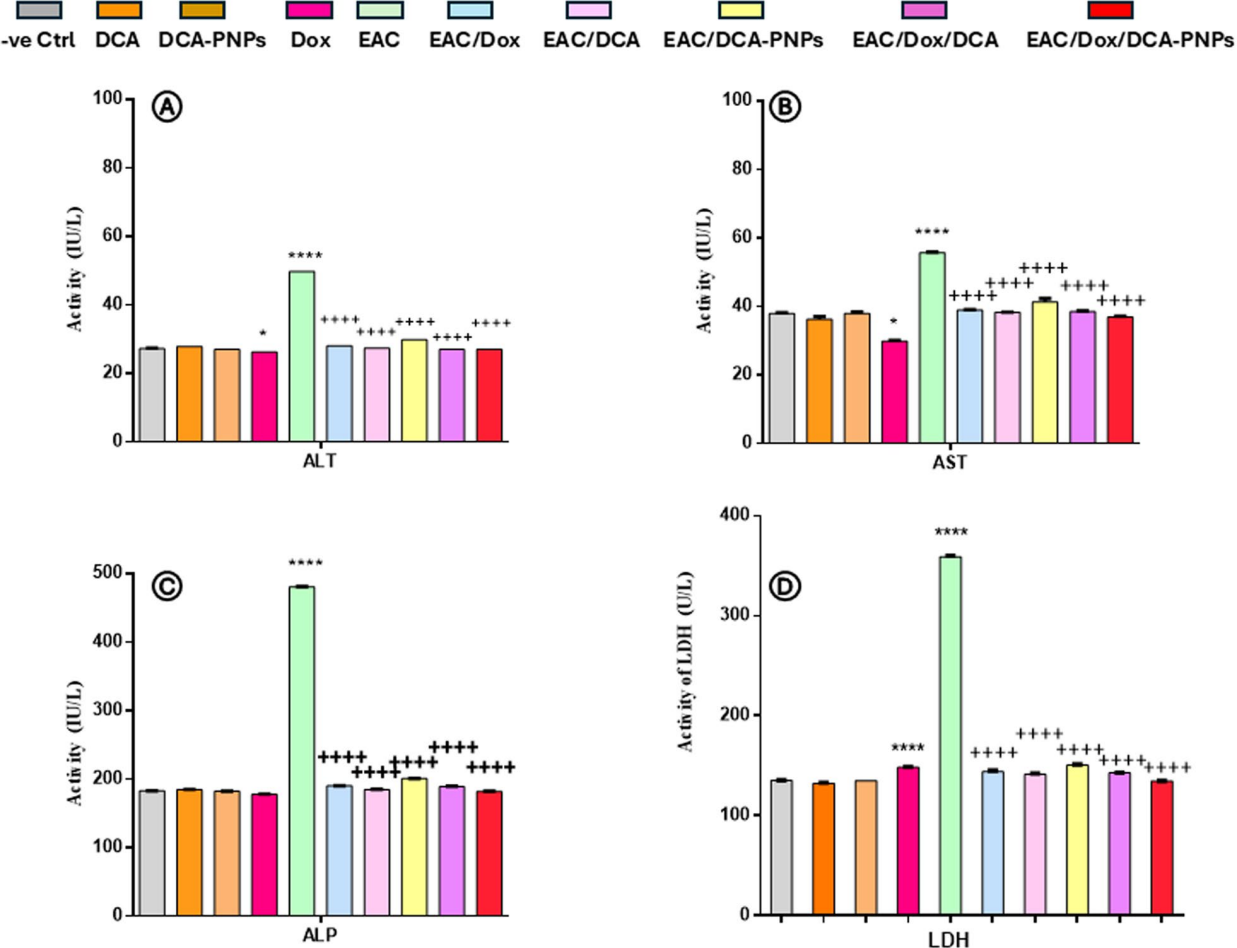
Serum ALT, AST, ALP, and LDH levels, data are presented as mean ± SE n = 4, (*p < *0.0001; p<0.05*) value: vs. control group, (^+^*p* < *0.0001*) value: vs. EAC-bearing group

**Fig. 2:**
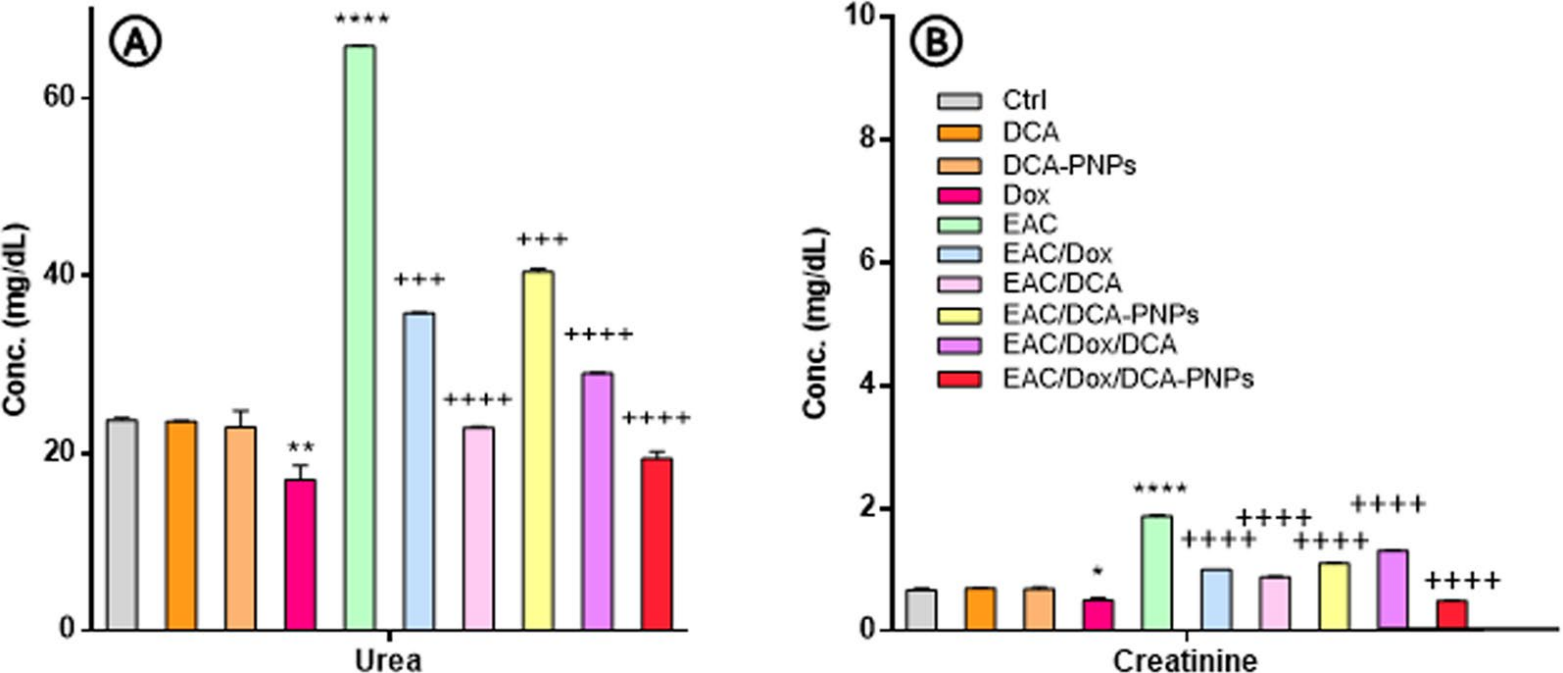
Serum urea and creatinine concentrations in all groups, data are presented as mean ± SE n = 4, (*p<0.05; *p*<0.01; *p* < *0.0001*) value: vs. control group, (^+^*p* < *0.0001*) value: vs. EAC-bearing group

Compared to Gp1, ALT, AST, ALP, creatinine, urea, LDH remained unchanged in Gp2 and Gp3. Gp4 showed decreased AST, ALP, creatinine, urea (-21, -3, -25, -28%; *p* < 0.0001), unchanged ALT, and increased LDH (10%; *p* < 0.0001). Gp5 showed increased ALT, AST, ALP, creatinine, urea, LDH (+ 83, + 47, + 163, + 75, + 177, 165%; *p* < 0.0001). Gp6-10 showed reduced levels versus Gp5 (*p* < 0.0001). Gp10's parameters indicated normal function: ALT 26.9 ± 0.15 IU/mL, AST 36.9 ± 0.2 IU/mL, ALP 181.7 ± 0.7 IU/L, creatinine 0.3 ± 0.02 mg/dL, urea 19.4 ± 0.8 mg/dL, LDH 134.65 ± 0.15 U/L (Figs. 1, 2).

### DCA-PNPs enhance the protein content and lipid profile

Compared to Gp1, TP, Alb, and Glb showed no change in Gp2 and Gp3. Gp4 showed decreased TP and Alb by −19 and −27% (*p* < 0.0001). Gp5 showed increased TP, Alb, and Glb by + 84, + 66, and + 103% (*p* < 0.0001). Versus Gp5, these parameters decreased in Gp6 to Gp10 (*p* < 0.0001). Gp10 showed: TP 6.01 ± 0.1, Alb 3.01 ± 0.06, Glb 3.01 ± 0.04 g/dL (−49, −41, −55%), (Supplementary Table 1). TC, TG, HDL-C, and LDL-C remained unchanged in Gp2 and Gp3 versus Gp1. Gp5 showed increased TC, TG, LDL-C (+ 182, + 429, + 200%), and decreased HDL-C (−23%) (*p* < 0.0001). Versus Gp5, HDL-C increased while TC, TG, LDL-C decreased in Gp6 to Gp10. Gp10 showed: TC 112 ± 0.6, TG 138 ± 1.2, LDL-C 50.7 ± 0.2, HDL-C 39.1 ± 0.1 mg/dL (−65, −82, + 32, −64%), (Supplementary Table 2).

### DCA-PNPs restore hepatic and renal oxidative-stress levels and increased antioxidants levels

**Fig. 3:**
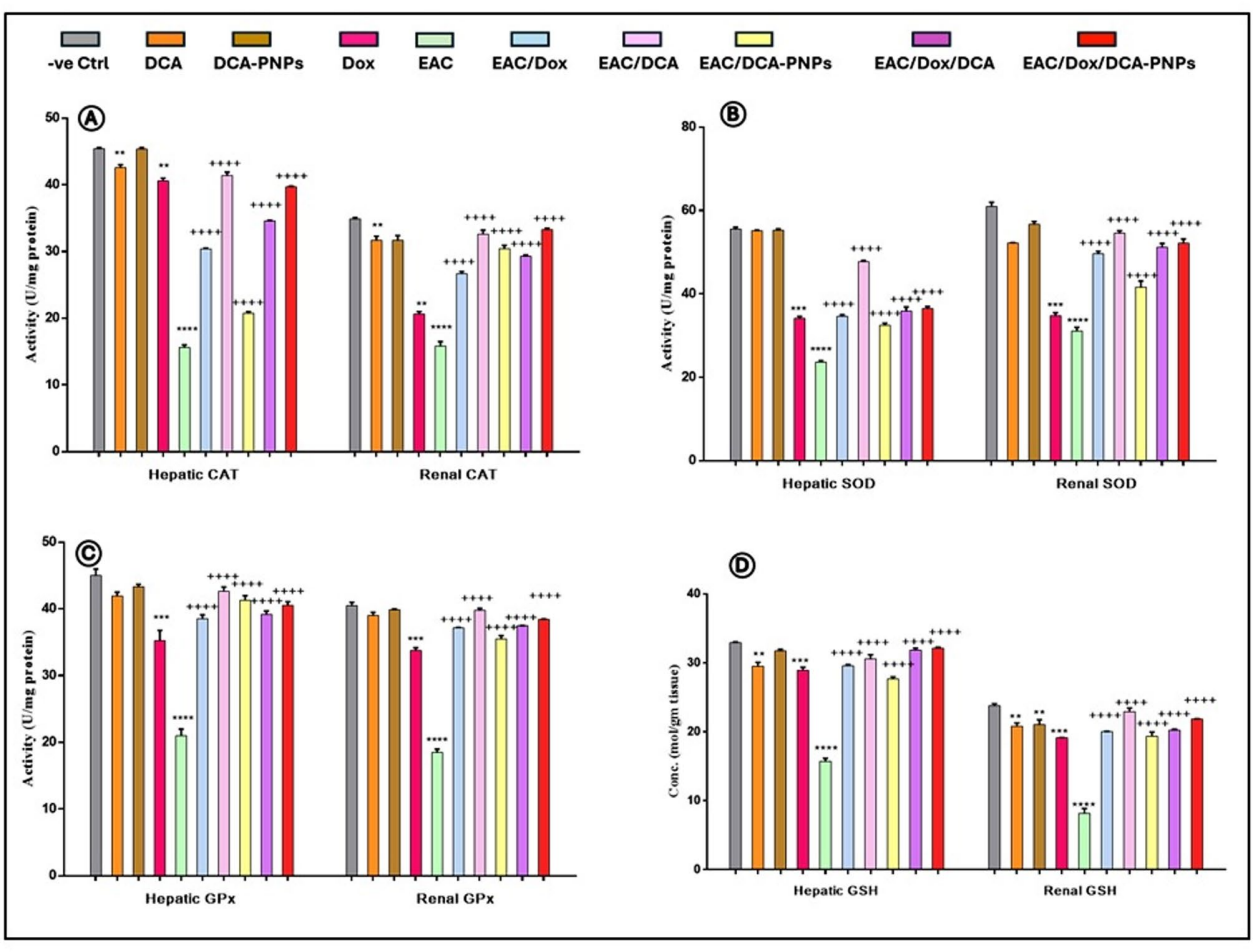
Hepatic and renal antioxidant parameters, **A** CAT, **B** SOD, **C** GPx, and **D** GSH levels in all groups, data are presented as mean ± SE n = 4, (*p<0.01; *p* < *0.0001*) value: vs. control group, (^+^*p* < *0.0001*) value: vs. EAC-bearing group

Compared to control (Gp1), hepatic/renal MDA/NO levels were unchanged in DCA (Gp2) and DCA-PNPs (Gp3) treated mice. Dox-treated mice (Gp4) showed unchanged hepatic/renal MDA but increased in NO (+ 29%, + 33.5%), respectively, (*p* < 0.0001). EAC mice (Gp5) showed increased hepatic/renal MDA/NO represented as + 321%, + 267.7% / + 178%, + 193%, respectively, (*p* < 0.0001). Compared to Gp5, hepatic/renal MDA/NO levels decreased in EAC mice treated with Dox (Gp6), DCA (Gp7), DCA-PNPs (Gp8), Dox/DCA (Gp9), and Dox/DCA-PNPs (Gp10), with Gp10 showing maximum decrease as -76%, -66% and -72.6% -63.8% respectively, (Supplementary Fig. 4). Hepatic/renal levels of SOD/GPx remained unchanged in Gp2-3, while hepatic/renal CAT/GSH decreased in Gp2, and only renal GSH decreased for Gp3. Gp4-5 showed decreased hepatic/renal SOD, CAT, GPx, and GSH. Versus Gp5, Gp6 showed lower hepatic/renal SOD/CAT but higher hepatic/renal GPx/GSH, while Gp7-10 showed increased levels, with Gp 9 and 10 showing optimal changes as in Gp 10 (+ 66%, + 25%, + 26%, + 109% for liver) and (+ 85%, + 110%, + 127%, + 180% for Kidney) respectively, and in Gp 9: (+ 64%, + 16%, + 25%, + 104% for liver) and (+ 76%, + 85%, + 108%, + 147% for Kidney) respectively (*p* < 0.0001) (Fig. 3).

## Discussion

The study investigated Dox/DCA-PNPs' therapeutic potential in EAC-bearing mice, examining effects on liver, kidney function, lipid profile, and oxidative-stress markers. Dox/DCA-PNPs treatment significantly improved biochemical parameters compared to conventional chemotherapy. The DCA-PNPs formulation may enhance Dox efficacy while targeting cancer cells and improving uptake [43, 44]. High NPs %EE and %DL values demonstrate effective drug encapsulation [45]. TEM and SEM imaging are used to observe morphology, size, and distribution. Their consistent size and shape indicate optimal nanoparticle synthesis and loading [46]. DCA-PNPs formulated with PLGA/PVA demonstrated stable properties, with increased %EE, %DL, and uniform size facilitating intracellular absorption and therapeutic effectiveness, aligned with previous study [47].

Dox alone reduced AST and creatinine, showing dose-dependent cryoprotection [48]. Research by [49] supports DCA's role in improving hepatic metabolism during sepsis. In EAC-bearing mice, liver tests show elevated serum AST, ALT, and ALP levels [50, 51, 79], indicating hepatic damage. Kidney tests reveal increased urea and creatinine, suggesting renal impairment [50]. Our study showed elevated enzymes, indicating organ damage from tumor progression. This aligns with [3]**,** showing cancer growth disrupts tissue architecture. Liver and kidney markers improved in treated groups, with EAC/Dox/DCA-PNPs being most effective, suggesting protection through reduced oxidative-stress [10, 52]. This corresponds with [53], showed DCA enhances PDH activity in diabetic rats. In EAC models, elevated serum LDH indicates tissue damage [54]. The study showed increased LDH in EAC-bearing mice and reduction in treated groups, particularly with Dox/DCA-PNPs [55–57]. Dox decreased organ markers but increased LDH, indicating partial protection but myocellular damage. This matches reports of Dox's cardiotoxicity through pro-oxidant effects [5]. Dox/DCA or PNPs reversed alterations towards normal levels. PNPs enhance tumor targeting [7], and DCA protects tissues from Dox-induced toxicity [10]. Improved organ function in Dox/DCA-PNPs group stems from targeting and mediated protection.

TP, Alb, and Glb levels indicate liver function, immune status, and cancer cell proliferation [58]. EAC increased serum protein and albumin due to inflammation and cytokine release [3, 40]. EAC-bearing mice had elevated TP, Alb, and Glb, correlating with increased serum proteins [3]. Hyperglobulinemia linked with tumor IL-6 overproduction [59]. Treatments reduced protein levels versus EAC group, with Dox/DCA-PNPs normalizing levels, supporting nanoparticle delivery's anti-inflammatory enhancement [8]. Antioxidants modulate serum proteins [60]. Cancer cell proliferation links to altered lipid profiles, with high TC, TG, LDL-C and low HDL-C reflecting metabolic syndrome [61]. EAC-bearing mice showed higher TC, LDL-C, and TG than normal group [9]. Treated groups improved, with EAC/Dox/DCA-PNPs most effective. These findings aligned with DCA's lipid-regulatory effects through AMPK activation [62–64]. NPs improve lipid metabolism [7], suggesting Dox and DCA-PNPs restore lipid homeostasis.

Oxidative-stress contributes to cancer development and chemotherapeutic toxicity, with MDA and NO indicating lipid peroxidation [65–67]. In EAC cells, oxidative-stress occurs when ROS production exceeds antioxidant capacity [68, 69] aiding tumor growth [70]. EAC-bearing mice had elevated MDA and NO, indicating metabolic imbalance, consistent with [7, 71, 72]**,** showing cancer cells generate excess ROS, causing DNA damage. Dox treatment lowered MDA and NO by suppressing tumor growth [73, 74]. DCA and DCA-PNPs reduce MDA/NO by shifting glycolysis to oxidative-phosphorylation. DCA increases mitochondrial-ROS in cancer cells, inducing apoptosis [75]. Combination groups reduce oxidative-stress through Dox's DNA intercalation and DCA's metabolic disruption. Dox/DCA-PNPs showed the greatest reduction through enhanced tumor penetration [11]. Antioxidant parameters maintain redox homeostasis and protect against oxidative damage [76]. Control group with DCA showed decreased CAT, GPx, and GSH, while Dox caused significant drops in antioxidant levels [74]. In EAC-bearing mice, SOD, CAT, GPx, and GSH were significantly depressed [7]. EAC groups treated with Dox, DCA, and DCA-PNPs showed improvements in antioxidant levels, aligning with studies on DCA's enhancement of antioxidant enzyme activity [10, 11]. Increased oxidative-stress markers in treated mice suggest combination therapy reduces oxidative burden while enhancing antioxidant capacity [77–82]. Our results show DCA mitigates Dox-induced toxicity and improves metabolic profiles in an EAC model.

### Limitations and future perspectives

Conduct long-term studies on the treatment's effects on cancer types and human cancer cell lines. Explore molecular mechanisms of DCA-PNPs and Dox synergistic effects to pave the way for clinical translation.

## Conclusion

DCA-PNPs reduce Dox adverse effects while improving hepatorenal functions, restoring lipid and protein homeostasis, and enhancing antioxidant defences in EAC-bearing mice. These findings offer a promising strategy for managing EAC and improving breast cancer treatment outcomes with reduced toxicity.

## Supplementary Information

Supplementary material 1.

## Data Availability

The datasets used and/or analysed during the current study are available from the corresponding author on reasonable request. Some of these data are included in this published article and its supplementary file.

## Abbreviations

Alb: Albumin
ALP: Alkaline phosphatase
ALT: Alanine transaminase
ANOVA: Analysis of variance
AST: Aspartate transaminase
CAT: Catalase
Create: Creatinine
DCA: Dichloroacetate
DCA-PNPs: Dichloroacetate NPs
Dox: Doxorubicin
EAC: Ehrlich ascites carcinoma
ELISA: Enzyme-linked immunosorbent assay
Glb: Globulin
GP: Group
GPx: Glutathione peroxidase
GSH: Glutathione
HDL-C: High-density lipoprotein cholesterol
HPLC: High-performance liquid chromatography
i.p: Intraperitoneal
IACUC: Institutional Animal Care and Use Committee
LDH: Lactate dehydrogenase
LDL-C: Low-density lipoprotein cholesterol
MDA: Malondialdehyde
NO: Nitric oxide
NPs: Nanoparticless
PBS: Phosphate buffer saline
ROS: Reactive oxygen species
SE: Standard error
SOD: Superoxide dismutase
TC: Total cholesterol
TG: Triglycerides
TP: Total protein
