# Supplementary material for: Dichloroacetate nanoparticles and doxorubicin combinatorial treatment augment the hepato-renal function in Ehrlich ascites carcinoma cells
Source: BMC Res Notes. 2025 Nov 13;18:481. doi: 10.1186/s13104-025-07534-3 (PMC12616949; doi:10.1186/s13104-025-07534-3)
Supplement: Supplementary file 1 — Supplementary material 1. [file 13104_2025_7534_MOESM1_ESM.pdf]

Supplementary file

Dichloroacetate nanoparticles and doxorubicin combinatorial treatment augment the hepato-renal function in Ehrlich ascites carcinoma cells.

Amira T. Khattab[1,2], Mai M. El-Keiy[2], Doha M. Beltagy[1], Maha M. Salem[2]*

[1] Biochemistry Department, Faculty of Science, Damanhour University, Damanhour 22514, Egypt

[2] Biochemistry Division, Chemistry Department, Faculty of Science, Tanta University, Tanta 31257, Egypt

E-mails: amira.khattab@sci.dmu.edu.eg, mai.ibrahim@science.tanta.edu.eg, dohabeltagy@sci.dmu.edu.eg, maha_salem@science.tanta.edu.eg,

Running title: Dichloroacetate nanoparticles and doxorubicin enhance hepato-renal profile in EAC cells.

***Correspondence**

Dr. Maha M. Salem,

Biochemistry Division, Chemistry Department, Faculty of Science, Tanta University, Tanta 31257, Egypt.

E-mail: maha_salem@science.tanta.edu.eg

ORCID ID: 0000-0002-9108-6932

Supplementary Table 1: Serum protein content of different treated groups.

Groups	TP (g/dL)	Alb (g/dL)	Glb (g/dL)
**-ve control**	6.4±0.1	3.06±0.1	3.3±0.04
**Ctrl/DCA**<br>**% Change**	6.45 ±0.35<br>1 %	3.72±0.02<br>21 %	2.73±0.37<br>-18 %
**Ctrl/DCA-PNPs**<br>**% Change**	5.9± 0.3<br>-8 %	3.2±0.1<br>4 %	2.7±0.2<br>-19 %
**Ctrl/Dox**<br>**% Change**	5.18 ±0.3*<br>-19 %	2.2±0.15*<br>-27 %	2.93±0.16<br>-12 %
**EAC**<br>**% Change**	11.8±0.1*<br>84	5.1±0.8*<br>66	6.7±0.7*<br>103
**EAC/Dox**<br>**% Change**	6.9±0.2^{+}<br>-41	3.4 ± 0.2^{+}<br>-33	3.5±0.3^{+}<br>-47
**EAC/DCA**<br>**% Change**	6.7±0.1 $^{+}$<br>-43	3.3±0.1^{+}<br>-35	3.4±0.2 $^{+}$<br>-49
**EAC/DCA-PNPs**<br>**% Change**	6.3±0.25^{+}<br>-46	3.2±0.035^{+}<br>-37	3.1±0.28 $^{+}$<br>-53
**EAC/Dox/DCA**<br>**% Change**	6.4±0.02^{+}<br>-45	3.1± 0.05^{+}<br>-39	3.3±0.03^{+}<br>-50
**EAC/Dox/DCA-PNPs**<br>**% Change**	6.01±0.1^{+}<br>-49	3±0.06^{+}<br>-41	3±0.04^{+}<br>-55

Data are presented as mean ± SE n=4, (*$p<0.0001$) value: vs. control group, ($^{+}p<0.0001$) value: vs. EAC-bearing group.

Supplementary Table 2: Serum lipid profile of different treated groups.

Groups	TC (mg/dL)	HDL-C (mg/dL)	LDL-C (mg/dL)	TG (mg/dL)
**-ve control**	114.4±0.5	38.5±0.3	47.3±0.3	143±0.25
**Ctrl/DCA**<br>**% Change**	114.3 ±0.2<br>0 %	38.8 ±0.2<br>1 %	47±0.5<br>-1 %	142.7±0.2<br>0 %
**Ctrl/DCA-PNPs**<br>**% Change**	112.9± 0.1<br>-1 %	37.5 ±0.6<br>-3 %	46.66±0.6<br>-1 %	143.7±0.4<br>0 %
**Ctrl/Dox**<br>**% Change**	100 ±0.4*<br>-12 %	29.6± 0.4*<br>-23 %	44.2±0.07<br>-6 %	132.5±0.5<br>-8 %
**EAC**<br>**% Change**	323.1±1*<br>182	29.6±0.3*<br>-23	141±1.6*<br>200	757±1.5*<br>429
**EAC/Dox**<br>**% Change**	132±0.5+<br>-59	34.4±0.3+<br>16	67.5±0.1+<br>-52	153±0.2+<br>-80
**EAC/DCA**<br>**% Change**	147±0.1+<br>-45	32.6±0.4+<br>10	83.2±0.3+<br>-41	160±0.5+<br>-79
**EAC/DCA-PNPs**<br>**% Change**	120±0.3 +<br>-63	36.7±0.3+<br>24	55±0.6+<br>-61	145±0.1+<br>-81
**EAC/Dox/DCA**<br>**% Change**	131.1±1+<br>-59	35±1.1+<br>18	65.6±1 +<br>-54	151± 0.6+<br>-80
**EAC/Dox/DCA-PNPs**<br>**% Change**	112±0.6+<br>-65	39.1±0.1+<br>32	50.7±0.2 +<br>-64	138±1.2+<br>-82

Data are presented as mean ± SE n=4, (*$p<0.0001$) value: vs. control group, ($^{+}p<0.0001$) value: vs. EAC-bearing group.

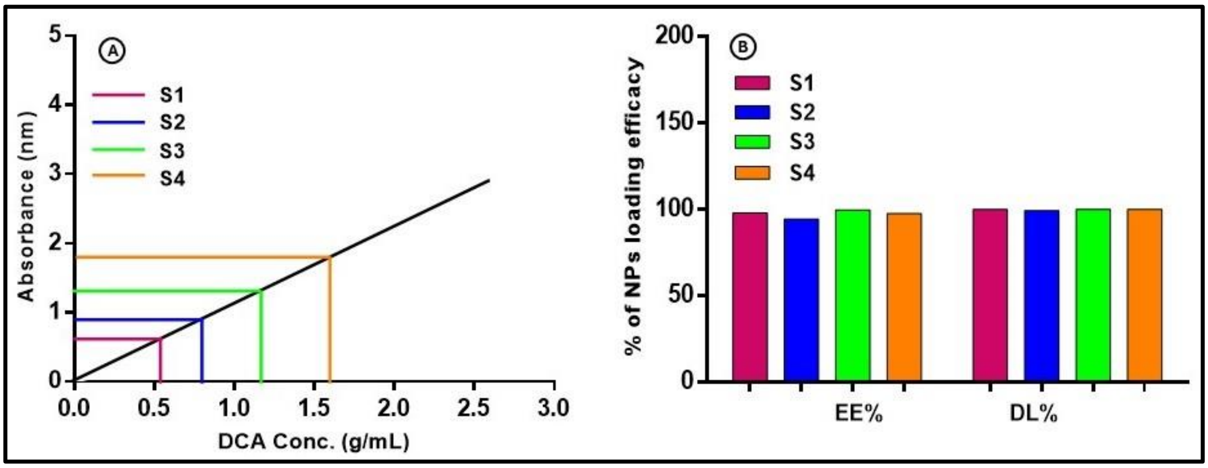

Supplementary Figure 1: (A) The calibration curve of DCA-PNPs, **(B)** the DCA-PNPs %EE and %DL.

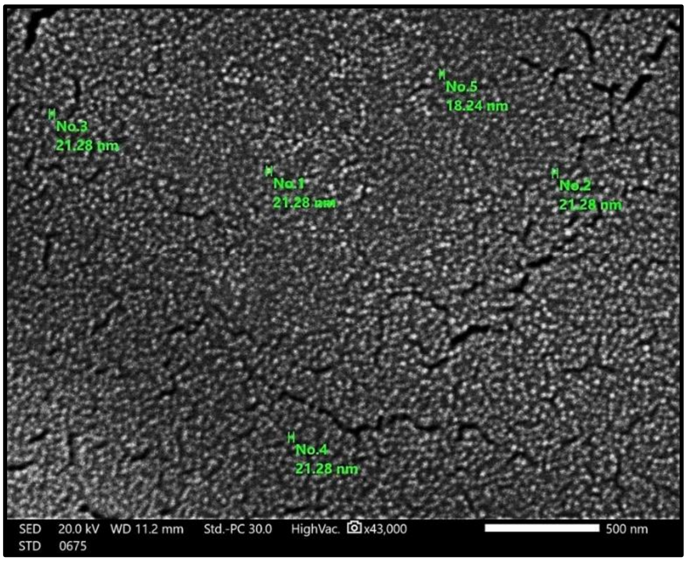

Supplementary Figure 2: SEM image illustrating the surface properties of DCA-PNPs.

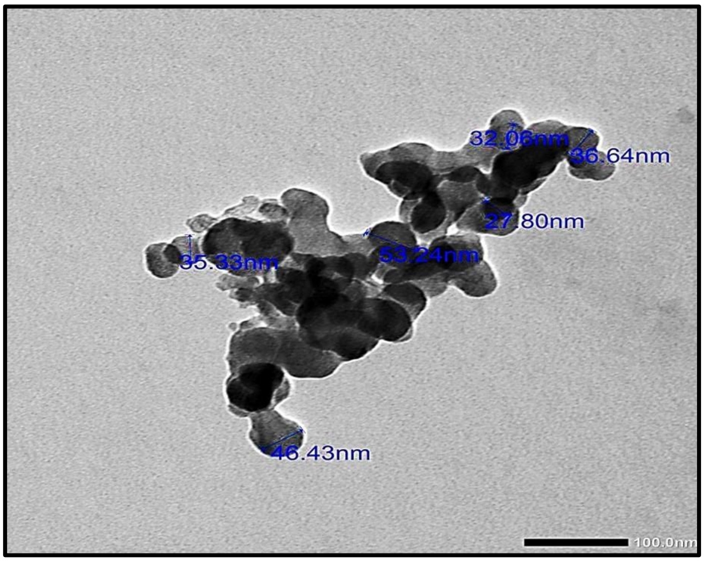

Supplementary Figure 3: TEM picture demonstrating the dimensions of DCA-PNPs.

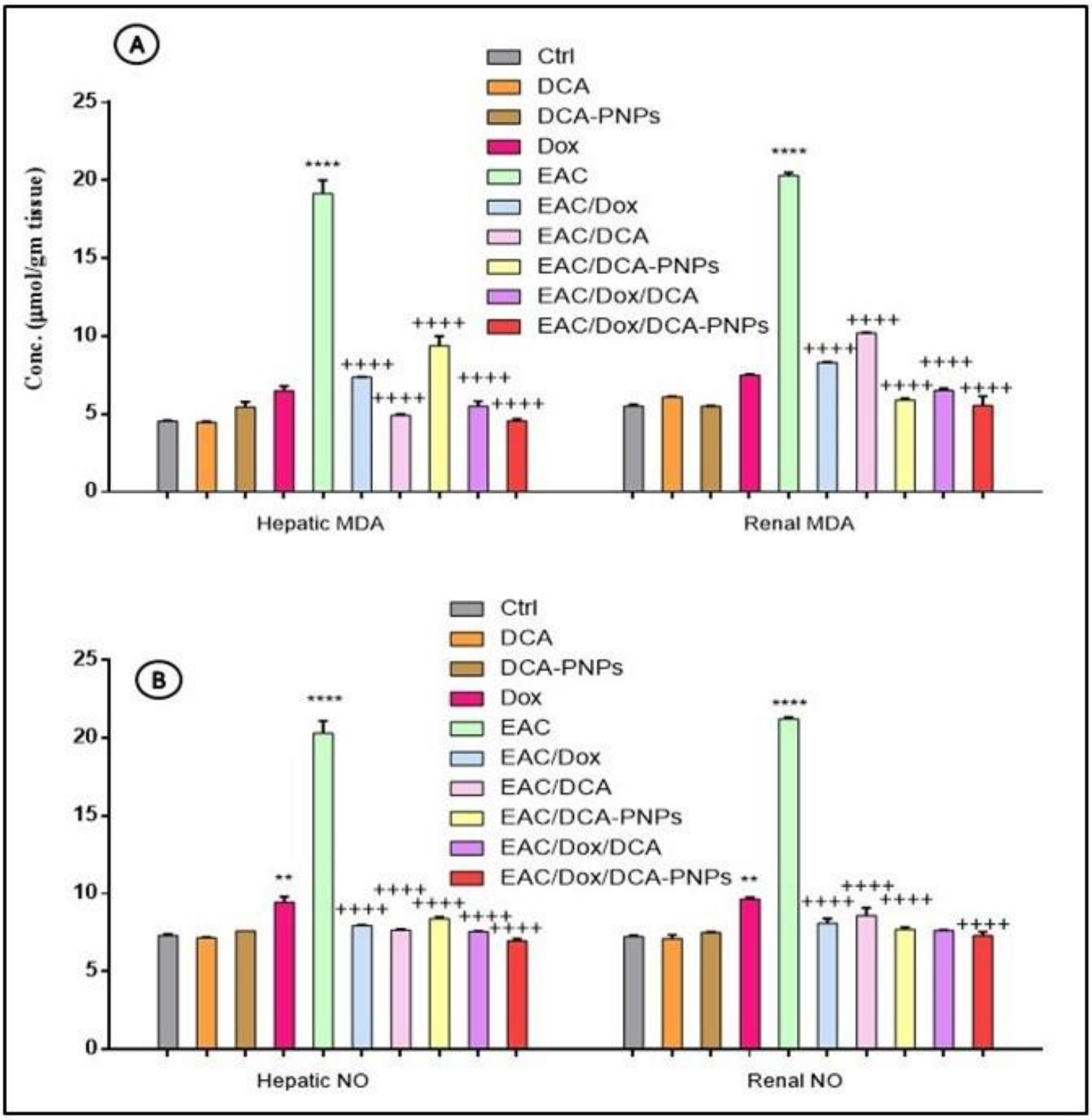

Supplementary Figure 4: Hepatic and renal oxidative stress parameters, **A:** MDA and **B:** NO concentrations in all groups, data are presented as mean ± SE n=4, (*$p<0.0001$) value: vs. control group, ($^{+}p<0.0001$) value: vs. EAC-bearing group.

Ethical approval (Arabic) Statement

كلية العلوم

جامعة طنطا

محضر اجتماع لجنة أخلاقيات البحث العلمى

يوم الثلاثاء ٢٠٢١/٢/٩

اجتمعت لجنة أخلاقيات البحث العلمي يوم الثلاثاء الموافق ٢٠٢١/٢/٩ الساعة الثانية عشر ظهرا برئاسة :

السيد الأستاذ الدكتور/ يحيي عبد الجليل محمود وكيل الكلية للدراسات العليا والبحوث وبحضور كل من :

أ.د/ طارق مصطفى محمد	عميد الكلية أستاذ بقسم الكيمياء
أ.د/ السيد ابراهيم السيد سالم	أستاذ بقسم علم الحيوان
أ.د/ محمد لبيب سالم	أستاذ بقسم علم الحيوان
أ.د/ صبرى على عبدالله النجار	أستاذ بقسم علم الحيوان
أ.د/ هاله فوزى حسن	أستاذ بقسم الكيمياء
أ.د/ على سليمان على	أستاذ بقسم جيولوجيا
أ.د/ جيهان احمد اسماعيل	أستاذ بقسم النبات
السيدة / نوال احمد علوان	امينا للجنة

ولم يحضر كلا من :-

السيد الاستاذ الدكتور/ بهجت يوسف البرادعي

السيد الاستاذ الدكتور/ مدحت احمد الدمسيسى

وذلك لمناقشة الموضوعات التالية :-

الموضوع رقم ١ :

التصديق على محضر الجلسة السابقة المنعقدة بتاريخ ٢٠٢١/١/٩ م .

****

الموضوع رقم ٢:

البروتوكول المقدم من الطالبة/ إميره طه احمد خطاب قسم الكيمياء

بعنوان : Role of nano- dichloroacetate on pyruvate dehydrogenase kinase, and its synergistic effect with chemotherapy on experimental breast cancer .

القرار: تم اعتماد الإجراءات التى سوف تتخذها الباحثة نحو إجراء العينات وأخذ رقم للاعتماد :

IACUC-SCI-TU-0210

****

الموضوع رقم ٣:

البروتوكول المقدم من الطالبة/ فايزة مهيب كمال شومان قسم علم الحيوان
